# On the Use of Ergot

**Published:** 1841-11

**Authors:** George Fife


*On the Use of Ergot.* By George Fife, M. D.—Having long been convinced of the value of the ergot of rye as a medicine, I now beg to offer the following remarks to my professional brethren; premising, that what I am about to advance is not the offspring of any fanciful notions as to its *possible* effects, but a simple statement of the results by which its use has been followed in my practice for several years. This assurance seems to be especially necessary at the present time, when it is but too much the fashion to render facts subservient to hypotheses. Whatever, then, may be advanced in this communication has been actually observed: and I am perfectly satisfied (due discrimination being exercised) will be fully sanctioned by further experience.

To enter upon the consideration of the action of the ergot on the parturient uterus, or the very interesting and important question of its power to excite uterine action, is foreign to my present object; I shall therefore proceed at once to a statement of its efficacy as a medicine, and the diseases in which I deem it worthy of confidence.—They are—polypus uteri, attended with profuse hemorrhage; menorrhagia, where there is no inordinate action of the heart or arteries, or morbid sensibility of the uterine system; in leucorrhœa, when independent of inflammatory action; in chlorosis with amenorrhœa; and in dysmenorrhœa: in all of which cases I have had numerous opportunities of ascertaining its efficiency.

The first time I saw it exhibited as a medicine was by my lamented friend, the late Mr. Parr, of this town, so long ago as the year 1828. It was in a case of very large polypus of the uterus, accompanied with frequent and frightful attacks of hæmorrhage. He gave the ergot after all ordinary treatment had proved unavailing. The effect produced was not only the moderation of the hæmorrhage, but also the expulsion of large and numerous masses of the tumour, in many of which a distinct fibrous structure was perceptible. After its continued employment, the woman, who had arrived at the climacteric period, enjoyed comparative health and comfort, being freed from the repeated and alarming hæmorrhages, and experiencing no inconvenience from the small portion of the tumour which, when last examined, still remained. To this case I owe the idea that it might possibly be useful in others. How far this has been justified, the sequel must decide.

In menorrhagia, by which term I do not mean the mere increased menstrual discharge, I have found this medicine of the greatest value. In this disease, however, it is necessary to ponder well on the individual circumstances of each case, and to use the utmost caution in ascertaining the cause on which the disease depends; as where such precaution has been neglected, it has been my lot to see both the sufferings and danger aggravated by the ergot. In this respect it does not differ from other active medicines. The slightest reflection will suffice to call to mind the very different states of the system in which this disease occurs. It may, for example, happen in the most phlogistic and plethoric, or it may arise in a person of a diametrically opposite constitution. If given in the first state it is decidedly prejudicial, unless preceded by such means as are calculated to remove alike the plethora and morbid sensibility; and even when this has been done, its operation requires to be carefully observed. In the last it acts more beneficially, as it at once raises the nervous energy of the uterus, and through this medium probably imparts increased tone to the relaxed and debilitated vessels from which the exhalation takes place. The following case affords a striking example of the utility of the medicine:

In 1834 I was requested to visit a poor woman who had for many years led a most abandoned life, and who was apparently in a dying state from extreme exhaustion. Her age might be about thirty-four. On inquiry, I learnt that for the two preceding years she had suffered from repeated and severe hæmorrhages from the uterus, and that, on the present occasion, the discharge had continued for many days, until, when I saw her, though still abundant in quantity, it was not more colored than the serum of healthy blood. In short, she seemed to be rapidly sinking. I ordered her immediately a full dose of the acetate of lead, with opium, to be repeated every four hours; and port-wine or brandy, in sago, to be given frequently. Cold was applied, both externally and internally, by means of an injection of a strong solution of alum. For a brief space of time she appeared somewhat relieved; but in two or three days the hæmorrhage recurred with increased violence, the fluid also being more sanguineous. Having in other cases, fancied that the ergot had been of use, I immediately gave her ʒss. of the powder, to be repeated in ℈i. doses every three or four hours. From the lapse of an hour after the first dose she experienced manifest relief; and on seeing her in the evening, I directed the third dose not to be given till the sixth hour, unless any recurrence of the discharge should render it necessary. Next day she continued better; and the medicine was directed to be taken only night and morning. From this visit she went on gradually improving; and for two years after, when I had occasion to prescribe for her, she had experienced no return of hæmorrhage. She appeared quite well in health, but completely blanched in colour. To this I might add several other cases, occurring in persons of asthenic constitution, in which similar good effects were derived from the ergot, but consider that it would only be a needless occupation of your columns, and your readers' time. It may be enough to say, that seven years of subsequent practice have confirmed my reliance on the ergot in such cases. Even where an opposite diathesis prevails it has been employed advantageously, combined with conium and hyoscyamus. In such persons it is advisable to premise a moderate abstraction of blood from the system, and to keep up a depletory action by means of saline cathartics. In these cases, however, the infusion of roses with sulphuric acid and digitalis, or alum, seem more appropriate.

In leucorrhœa it has been highly useful. In this disease, as in menorrhagia, it is necessary to ascertain the state of the system, more particularly the uterine disorder on which it depends; as where any degree of inflammation is present it will be injurious. In several distressing cases of this disease, where the strongest astringent injections had been employed without any effect, except exciting inflammatory action which did not previously exist, I have found the ergot, aided by injection of simple warm water, or the decoction of poppy capsules, perfectly successful. I may observe *en passant* that from considerable opportunities of observation, it is my firm belief that in a vast proportion of cases of lucorrhœa, stimulant and astringent injections are not only uncalled for, but absolutely contra-indicted; at the same time, it must be acknowledged, that they are occasionally of great use.

In chlorosis and amenorrhœa I have frequently experienced the good effects of the ergot, after aloes, iron, valerian, cantharides, &c., had all been employed without the slightest advantage. In these cases, when extreme nervous susceptibility exists, it may be most advantageously combined with the valerian, and where the alvine system is torpid, with aloes. When aloes are employed, I attach the preference to the Barbadoes: in doing so I may be somewhat empirical, as it is difficult to account for the superiority of the Barbadoes over the socotrine, as an emenagogue, unless it be ascribed to the larger quantity of bitter principle contained in the former.

In dysmenorrhœa the ergot has been most useful. In one case, accompanied with what may without exaggeration be termed torture at each period, it appeared almost magical in its operation. It is right to state that it was given in combination with the valerian; which medicine, however, had been previously given, but without any apparent benefit. To enter into the detail of individual cases is not only tedious, but would also occupy too much of your valuable columns. I shall therefore endeavor, with the utmost possible brevity, to explain the apparent paradox of a medicine being of use in cases at first sight so pathologically opposite.

It is well known that the same cause operating on different constitutions will be followed by effects proportionately variable. This is a fact which does not admit of refutation, whether it be applied to a man morally or physically. So then it is with regard to the uterine system, as every man of moderate experience will admit that the same morbid condition will, according to peculiarity of constitution, produce a very dissimilar train of symptoms. Thus, whilst a congested state of the uterine vessels will in one person lead to menorrhagia, the same cause operating on a different constitution will be attended with leucorrhœa. Again, when the nervous system of the uterus is at fault, we in one person have chorea, in another simple hysteria, in others cardiac and pulmonic symptoms, &c. &c. This is especially true in regard to menorrhagia and leucorrhœa. These diseases may arise alike in the sthenic and asthenic diatheses, which must of course render a corresponding modification of our remedial measures necessary. Without using much discrimination, we aggravate rather than alleviate the suffering inseparable from disease, and enrol ourselves under the banner of empiricism.

I have merely to add, that I consider myself fully justified in recommending the ergot as an excellent emenagogue and anti-hæmorrhagic agent. Should this recommendation be followed by beneficial results in the hands of other professional men, I will regard myself as amply rewarded for the trouble of putting together the foregoing remarks, the correctness of which can only be proved by trials more extensive than private practice; besides which, every man is liable to view with *partiality* any plan of treatment, which, without authority, he adopts and finds successful.

P. S. The doses in which I have prescribed it, have varied from gr. x. to ℈i. of the powder, ʒss. to ʒi. of the concentrated tincture. A good formula for decoction is given in Foot's Medical Almanack, although it appears to me that the quantity of ergot is rather small.

My friend, Mr. Bennet of Gateshead, informs me that he has given the ergot in a case of hæmoptysis with very satisfactory results.—*Lon. Med. Gaz.*

